# Exploring lemology teaching with “internet plus” flipped classroom pedagogy

**DOI:** 10.1186/s12909-023-04309-x

**Published:** 2023-05-16

**Authors:** Yu-Xin Cao, Shu-Lin Xia, Zheng-Yun Zhu, Fan-Rong Zeng, Hai-Ning Li, Ting-Ting Zhang, Yong-Juan Liu

**Affiliations:** 1 Jinzhou Medical University, Jinzhou, 121001 China; 2 Department of Infectious Disease, Affiliated Taixing People’s Hospital of Yangzhou University, Taixing, 225499 China; 3 Department of Infection, The First Affiliated Hospital of Kangda College of Nanjing Medical University, The First People’s Hospital of Lianyungang, No. 6 Zhenhua Road, Haizhou District, Lianyungang, 222002 China; 4 Department of Medical Technology, Kangda College of Nanjing Medical University, Lianyungang, 222000 China; 5 Central laboratory, The First Affiliated Hospital of Kangda College of Nanjing Medical University, The First People’s Hospital of Lianyungang, No. 6 Zhenhua Road, Haizhou District, Lianyungang, 222002 China

**Keywords:** Flipped classroom, Internet, Lemology

## Abstract

**Background:**

To investigate the use of flipped classroom pedagogy based on “Internet plus” in teaching viral hepatitis in the lemology course during the COVID-19 epidemic.

**Methods:**

This study included students from the clinical medicine general practitioner class at Nanjing Medical University’s Kangda College, with the observation group consisting of 67 students from the 2020–2021 school year and the control group consisting of 70 students from the 2019–2020 school year. The observation group used “Internet plus” flipped classroom pedagogy, while the control group used conventional offline instruction. The theory course and case analysis ability scores from the two groups were compared and analyzed, and questionnaire surveys were administered to the observation group.

**Result:**

After the flipped classroom, the observation group had significantly higher theoretical test scores (38.62 ± 4.52) and case analysis ability scores (21.08 ± 3.58) than the control group (37.37 ± 2.43) (*t =* 2.024, *P* = 0.045) and (19.16 ± 1.15) (*t =* 4.254, *P* < 0.001), respectively. The questionnaire survey in the observation group revealed that the “Internet plus” flipped classroom pedagogy approach can help enhance students’ enthusiasm to learn, clinical thinking ability, practical application ability, and learning efficiency, with satisfaction rates of 81.7%, 85.0%, 83.3%, and 78.8%, respectively; 89.4% of students expressed hope that whenever physical classes resumed, the offline courses could be combined with this pedagogy approach.

**Conclusion:**

The use of the “Internet plus” flipped classroom pedagogy technique for teaching viral hepatitis in a lemology course boosted students’ theory learning ability as well as their case analysis ability. The majority of students were pleased with this type of instruction and hoped that whenever physical classes resumed, the offline courses may be integrated with the “Internet plus” flipped classroom pedagogical approach.

## Background

Lemology is a broad field that includes prevention, treatment, and health care. It is extremely useful in safeguarding people’s health and enhancing quality of life, especially when dealing with epidemics that threaten public health [1–3]. Most disorders associated with lemology, such as hemorrhagic fever with renal syndrome, epidemic cerebrospinal meningitis, and epidemic encephalitis B, have considerably lower incidences than those associated with internal medicine, surgery, gynecology, and pediatrics, making them readily misdiagnosed [4, 5]. People have increasingly recognized the importance of generating interest in medical students, delivering humanistic care-based instruction, and ensuring students can acquire basic theoretical information in this field, particularly in the aftermath of the COVID-19 pandemic [6, 7]. Educational informatization is steadily being used to change higher education and improve educational quality [8–10]. Many universities have gradually introduced a variety of online courses through massive open online course (MOOC) and live video platforms [11, 12]. We integrated the case-based teaching method (CBL) with the staged goal teaching method from July 2018 to July 2019 and produced good results when compared to routine teaching approaches. During the epidemic, we implemented flipped classroom in the undergraduate teaching of lemology utilizing the school’s online course platform, incorporating case studies and heuristic questions to pique students’ interest. The learning impacts were assessed, i.e., what was the teaching effect of the flipped classroom in lemology via the internet, and whether it has merit. We did an analysis based on the data and report the results as follows:

## Participants and methods

### Study participants

Participants in the study were clinical medicine general practitioner students from Nanjing Medical University’s Kangda College from the 2019–2021 cohort. The hospital’s ethics committee approved the study (Approval number: LW-20230323001-01), and the students were divided into observation and control groups using the random number table method. Inclusion criteria were as follows: (1) willing volunteers who were aware of the study’s topic in advance; and (2) students who completed the viral hepatitis lesson in the lemology course on time. The study included 136 students (73 males and 63 females aged 20 to 23 years), divided into 66 cases in the observation group and 70 cases in the control group. There were no significant differences between the two groups in enrolment theoretical scores and grade-point average scores. The gender and age distributions are displayed in Table 1.

**Table 1:** Comparison of general features between two groups of students

Groups	Number of cases	Gender (case)		Age (year)
		Male	Female	
Observation Group	66	37	29	21.50 ± 0.75
Control Group	70	36	34	21.68 ± 0.53
Statistics		0.293^a^		-1.681^b^
*P*		0.588		0.095

Note: ^a^chi-square test, ^b^t-test

## Teaching methods

The class was 3 h long and covered the viral hepatitis chapter of the 9th edition of lemology published by HUMAN HEALTH. The observation and control groups were taught by the same teachers to ensure that the study was not influenced by teacher quality. The students in both groups had the same major. The control group received routine offline instruction: one week before the class began, students were alerted to prepare for the class on their own. Theoretical lectures were delivered in the sequence of genesis, epidemiology, clinical symptoms, diagnosis, and therapy during the 3-hour class, and critical thinking questions were asked after class. The observation group used the “Internet plus” flipped classroom pedagogy technique, which was divided into three stages:

“Pre-class preparation” was the first stage. One week before the start of class, students were given preview courseware and exercises, as well as critical thinking questions:

(1) Short videos of 5 ~ 8 min were recorded as preview courseware for the required content, such as pathogen categorization, clinical symptoms and classification of viral hepatitis, and diagnostic criteria, according to the curriculum. Each short video presented a vital information point while simultaneously highlighting the key points and challenges. Considering the learning circumstances due to distance education, the content covered all stages of disease occurrence and progression as far as possible. (2) The preview tasks were case-based, with topics including acute hepatitis after HBV infection, hepatitis B carrier status, chronic viral hepatitis B, post-hepatitis B cirrhosis, and hepatitis B-related liver cancer. Furthermore, the following disease phases and kinds were covered: mild, moderate, and severe chronic hepatitis, as well as active, quiescent, compensated, and decompensated liver cirrhosis. (3) Critical thinking questions were added, as well as photographs of positive signs of common liver diseases, blood routine tests, liver function tests, and the etiology of hepatitis A, B, C, D, and E viruses, to assist students in identifying the disease, mastering positive symptoms, and correctly interpreting auxiliary test reports.

Eyebrow tattooing,” “tattooing,” and “tooth extraction” are common procedures. Micro-videos of the related operations were created to allow students to consider which diseases will spread based on the content of the viral hepatitis chapter, what would happen if these operations were not standardized and the instruments were not sterilized to the standard, and how to disinfect to avoid hepatitis virus infection. The internet-based flipped classroom allowed students to discuss their own ideas and deepen their visual sense of the transmission route.

The second stage was “flipped classroom implementation and knowledge comprehension.“ This stage was divided into three class hours according to the syllabus. This level began with standard cases and case discussions. Beginning with the three elements of infectious disease—the source of infection, the transmission route, and the susceptible population—the knowledge points of etiology, clinical manifestation, and diagnosis basis were gradually analyzed, leading to treatment principles and the formulation of specific treatment plans. In the instance of hepatic encephalopathy, for example: (1) In the event of an induction infection, anti-infection treatment should be administered on a timely, adequate, and complete basis. In the early stages of an infection, based on the patient’s immune status and clinical manifestations, it is necessary to analyze the possible infectious routes (respiratory tract, digestive tract, biliary tract, reproductive tract, and urinary tract) and pathogenic bacteria in order to empirically use relevant drugs. (2) In the event of ammonia intoxication, the following procedures should be followed: (a) low protein diet (protein intake < 0.5/kg.d); (b) intestinal bacteria inhibition (oral norfloxacin); (c) acidification and stool blockage (oral lactulose); and (d) blood ammonia neutralization (acetyl glutamine, ornithine aspartate, sodium glutamate). The treatment plan should be adjusted further based on the individual clinical conditions of each patient at the time. The students were divided into four groups and instructed by the lecturers to think about the questions, discuss them, and then choose 1–2 representatives to answer the questions, who were then supplemented by the other students. Classroom summary and Q&A: the lecturers summarized the conversations with the students, examined the correct answer rate of the classroom exercises, identified weak areas, and strengthened them with additional attention.

“Consolidation and extension” was the third stage. The lecturers set multiple choice test questions based on the students’ intelligence to consolidate the knowledge points, and then handed over the results to clinical professors who confirmed the data.

## Observation indicators

### (1) Classroom evaluation

To encourage students to actively connect with the classroom, we implemented pre-class scanning and signing-in, barrage engagement, PPT (Microsoft Office Power Point) synchronization, random roll call, and sending and receiving test questions in class to improve the efficiency of online lectures.

### (2) Comprehensive ability judgment

Questions were given to assess theoretical knowledge and case analysis ability. In the theoretical examination, 60 points were awarded for fundamental and specialized knowledge, while 40 points were assigned to case analysis questions. Higher scores may indicate higher information retention or mastery.

### (3) Questionnaire survey

After the training sessions, the questionnaires were disseminated and collected, and reliability and validity analyses were performed to optimize the questionnaires (Cronbach’s α: reliability coefficient was 0.929, and the validity coefficient was 0.796). Students were asked whether they felt more motivated to learn, if they had better clinical reasoning and practical application skills, if they felt they were learning more efficiently, and if they hoped that offline courses might be merged with this type of teaching whenever physical classes began. There were three answer options for each question: very satisfied, satisfied, and dissatisfied. The calculation formula for the total satisfaction rate is: Total satisfaction rate = “Very satisfied” cases + “satisfied” cases / total number of cases. A total of 66 valid questionnaires were collected, with an effective rate of 100%.

## Statistical methods

Statistical analysis was performed using SPSS20.0; continuous variables are represented as mean ± standard deviation; the independent sample t test was used to compare the two groups; and categorized variables are expressed as cases (percent); the chi-squared test was used to compare the two groups. *P* < 0.05 was considered statistically significant.

## Result

### Classroom evaluation

With the exception of the students who were unable to complete the course owing to network outage, 66 students in the observation group completed the scanning and sign-in on time. The course had more active real-time comment-based interaction. The instructor asked a total of 9 questions, and 594 replies were collected on the spot, with 590 accurate responses; the rate of correct classroom answers was 99.3%.

## Comparison of the comprehensive abilities of the two groups

The assessments of theoretical knowledge and case analysis ability of the two groups are displayed in Table 2. The observation group had a significantly higher theoretical test score (38.62 ± 4.52) and case analysis ability score (21.08 ± 3.58) than the control group (37.37 ± 2.43) (*t* = 2.024, *P* = 0.045) and (19.16 ± 1.15) (*t* = 4.254, *P* < 0.001), respectively.

**Table 2:** Comparison of comprehensive ability between two groups of students

Groups	Number of cases	Theory test (points)	Case analysis ability test (points)
Observation Group	66	38.62 ± 4.52	21.08 ± 3.58
Control Group	70	37.37 ± 2.43	19.16 ± 1.15
*t*		2.024	4.254
*P*		0.045	< 0.001

## Questionnaire results of observation group

Students in the observation group were handed the questionnaire after class, and the findings are reported in Table 3. The majority of students believed that the “Internet plus” flipped classroom pedagogy approach was beneficial in terms of increasing enthusiasm for learning, clinical thinking ability, practical application ability, and learning efficiency, and they hoped that after physical classes resumed, this pedagogy approach could be combined with offline courses. Table 3 shows the questionnaire for the “Internet plus” flipped classroom pedagogy mode that was given to the observation group.

**Table 3:** Questionnaire results of “Internet plus” flipped classroom teaching methods in the observation group

Item	Grading (case)			Total Satisfaction Rate (%)
	Very satisfied	Satisfied	Dissatisfied	
1. 1. Enhancement of learning initiative	42	13	11	81.7
2. 2. Improvement of clinical thinking ability	32	19	9	85.0
3. 3. Practical application ability	40	26	10	83.3
4. 4. Improvement in learning efficiency	34	20	14	78.8
5. 5. After physical classes resume, it is hoped that offline courses will be combined with this pedagogy approach	41	18	7	89.4

## Discussion

During the COVID-19 epidemic, we investigated the efficacy of flipped classroom pedagogy based on an internet platform in teaching viral hepatitis in the lemology course. The findings revealed that, when compared to traditional offline teaching, this pedagogy approach can improve students’ theoretical achievement and case analysis ability, and that the implementation of an “Internet plus” flipped classroom can assist students in analyzing problems, applying them in practice, and increasing enthusiasm for learning and learning efficiency. The overall satisfaction rate was better than 75%, and the pedagogy approach was well received by the majority of the students. There are currently few questionnaires that can properly investigate the impact of medical students’ learning in a blended learning setting. It is encouraging to see the recent publication of a questionnaire that can clearly and effectively reflect the learning effect in a blended learning setting (Blended Learning Questionnaire, BLQ) [13]. The observation group benefited significantly from this method in the following ways: online audio-visual resources improved learning efficiency, group activities increased understanding of knowledge points, and students improved their self-learning and focused learning ability through regular integration of online resources outside of school. These findings are consistent with the findings of our earlier questionnaire.

The flipped classroom balances what is taught inside and outside of the classroom: students learn independently in advance, and the classroom is no longer a place for information transfer, but rather for teacher-student interaction. The essence is to learn first and then teach. Lecturers can be not just knowledge imparters, but also knowledge guides and edifiers. By using students as the primary body and questions as the guidance, students’ knowledge systems become more complete and integrated [14]. Some studies have combined the development of the flipped classroom with CBL, WeChat, micro-class, and other ways to improve teaching [15–17]. It demonstrates that the flipped classroom teaching paradigm has been acknowledged. However, in the past, flipped classrooms were rarely used in the teaching of infectious diseases. Our teaching content begins by igniting students’ hunger for knowledge and progresses to mastery and application of knowledge, demonstrating the benefits of flipped classroom in the teaching of infectious diseases.

However, we found a large gap between the lowest and highest scores in the theory course and case analysis abilities in the observation group: the scores in the theory course of the observation group and the control group were (38.62 ± 4.52) and (37.37 ± 2.43), and scores in case analysis ability were (21.08 ± 3.58) and (19.16 ± 1.15), respectively, suggesting that “Internet plus” flipped classroom pedagogy may cause two-level differentiation of the test scores. Pre-class preparation and classroom implementation of the “Internet plus” flipped classroom place great demands on the quality of the teachers, as well as require a significant amount of time and energy. Teachers need to constantly adjust the teaching plan according to the learning circumstances, explore the advantages of internet-based teaching, minimize the gap between students with high and low scores, and maximize the benefits for students.

Furthermore, most courses at medical colleges and universities are currently taught by clinicians, but access to medical resources is limited and highly limited, particularly in the context of the current epidemic. The following significant issues confront such a pedagogy approach: There needs to be (1) a system of incentives, rewards, and punishments for the teaching staff; (2) a system to control the quality of internet-based teaching; and (3) a system of homogenous management to ensure a consistent level of instruction for all students. Our study does have a few limitations. Students were randomly assigned to either the flipped class group or the control group; however, as students in both groups were aware of their assignment, the survey’s questions were subject to judgment bias.

In conclusion, the flipped classroom pedagogy approach based on the internet platform was a useful attempt during this epidemic, and showed positive outcomes for increasing students’ theoretical understanding of the viral hepatitis chapter, their ability for analysis of cases, their interest in learning, and their capacity for clinical reasoning. At the same time, flipped classroom implementation increases teachers’ workload. However, given the relatively fixed teaching path followed by teachers, the pre-class preview videos created during the initial class, the classic case sharing in class, and the classroom interaction plan can be improved and reused in the subsequent real-time flipped classroom, reducing teachers’ workload. With the gradual enhancement of teachers’ incentive, reward, and punishment mechanisms, the viability of investing in educational resources is something to look forward to. Meanwhile, we will continue to enhance and optimize the teaching plan based on student feedback, and integrate this teaching technique with the offline course, as well as increase the teaching quality in the lemology course by constant investigation and refinement.

## Data Availability

All data generated or analysed during this study are included in this article. Further enquiries can be directed to the corresponding author.

## Abbreviations

MOOC: massive open online course
CBL: case-based teaching method

## References

[1] Zhang Li M, Shasha LC (2021). Teaching reform and practical exploration of integrating Courses Ideological and Political in Infectious Disease Course. China Continuing Medical Education.

[2] Sayıner AA, Ergönül E (2021). E-learning in clinical microbiology and infectious diseases. Clin Microbiol Infect.

[3] Rydel TA, Bajra R, Schillinger E (2021). Hands off yet all in: a virtual clerkship pilot in the ambulatory setting during the COVID-19 pandemic. Acad Med.

[4] Wang Q, Yue M, Yao P, Zhu C, Ai L, Hu D, Zhang B, Yang Z, Yang X, Luo F, Wang C, Hou W, Tan W. Epidemic Trend and Molecular Evolution of HV Family in the Main Hantavirus Epidemic Areas From 2004 to 2016, in P.R. China. Front Cell Infect Microbiol 2021 Feb 3;10:584814. doi: 10.3389/fcimb.2020.584814. 10.3389/fcimb.2020.584814 PMC7886990 33614521

[5] Li J. [Historical review of the epidemic situation, prevention and control of epidemic cerebrospinal meningitis during 1966 to 1967]. Zhonghua Yi Shi Za Zhi. 2020 Mar 28;50(2):101–109. Chinese. doi: 10.3760/cma.j.cn112155-20200223-00021. 10.3760/cma.j.cn112155-20200223-00021 32536104

[6] Cervantes J (2020). The Future of Infectious Diseases Education. Med Sci Educ.

[7] Sukumar S, Zakaria A, Lai CJ, Sakumoto M, Khanna R, Choi N (2021). Designing and implementing a novel virtual Rounds Curriculum for Medical students’ Internal Medicine Clerkship during the COVID-19 pandemic. MedEdPORTAL.

[8] De Ponti R, Marazzato J, Maresca AM, Rovera F, Carcano G, Ferrario MM. Pre-graduation medical training including virtual reality during COVID-19 pandemic: a report on students’ perception. BMC Med Educ. 2020;20(1):332. Published 2020 Sep 25. doi:10.1186/s12909-020-02245-8. 10.1186/s12909-020-02245-8 PMC7517753 32977781

[9] Jeffres MN, Kufel WD, Biehle LR (2019). A Comprehensive Survey of Infectious Diseases Curriculum among US Pharmacy Schools. Am J Pharm Educ.

[10] Ray JM, Wong AH, Yang TJ (2021). Virtual Telesimulation for Medical Students during the COVID-19 pandemic. Acad Med.

[11] Lee IR, Kim HW, Lee Y (2021). Changes in undergraduate medical education due to COVID-19: a systematic review. Eur Rev Med Pharmacol Sci.

[12] Chua WL, Ooi SL, Chan GWH, Lau TC, Liaw SY (2022). The Effect of a Sepsis Interprofessional Education using virtual patient Telesimulation on Sepsis Team Care in Clinical Practice: mixed methods study. J Med Internet Res.

[13] Ballouk R, Mansour V, Dalziel B, Hegazi I. The development and validation of a questionnaire to explore medical students’ learning in a blended learning environment. BMC Med Educ. 2022;22(1):4. Published 2022 Jan 3. doi:10.1186/s12909-021-03045-4. 10.1186/s12909-021-03045-4 PMC8722320 34980084

[14] Li Zhen G, Cheng C, Zhiqiao (2022). Application of flipped Classroom in Medical Education. China Continuing Medical Education.

[15] Brockhoff RA, Hicks SR, Salmanton-García J (2021). Training in infectious diseases across Europe in 2021 - a survey on training delivery, content and assessment. Clin Microbiol Infect.

[16] Zhou, Dan et al. Deng Wen-hui, Lv Qiu-bo,. Application of WeChat-based flipped classroom model in obstetrics and gynecology teaching of general practice residency training. Chinese Journal of General Practitioners. 2021;20(01):97–99.

[17] Qin Yong-ting, Chen L, Shanshan C (2019). Application of flipped Classroom based on micro-lecture in physiological experiment teaching of higher vocational nursing Specialty. Chin J Gen Pract.

